# A Novel PAA Derivative with Enhanced Drug Efficacy in Pancreatic Cancer Cell Lines

**DOI:** 10.3390/ph11040091

**Published:** 2018-09-22

**Authors:** Ali Alsuraifi, Paul Kong Thoo Lin, Anthony Curtis, Dimitrios A. Lamprou, Clare Hoskins

**Affiliations:** 1 Institute of Science and Technology in Medicine, Keele University, Keele ST5 5BG, UK; a.t.y.alsuraifi@keele.ac.uk (A.A.); a.d.m.curtis@keele.ac.uk (A.C.); 2 College of Dentistry, University of Basrah, Basrah 61004, Iraq; 3 School of Pharmacy and Life Sciences, Robert Gordon University, Aberdeen AB10 7GJ, UK; p.v.s.kong-thoo-lin@rgu.ac.uk; 4 School of Pharmacy, Queen’s University Belfast, Belfast BT9 7BL, UK

**Keywords:** nanomedicine, nanopharmaceutics, drug solubilisation, pancreatic cancer, bisnaphthalimide

## Abstract

Nanoparticles have been shown to be effective drug carriers in cancer therapy. Pancreatic cancer forms dense tumours which are often resistant to drug molecules. In order to overcome such multidrug resistance, new drug entities, novel delivery systems and combination therapy strategies are being explored. In this paper, we report the design and synthesis of a poly(allylamine)-based amphiphile modified with hydrophobic naphthalimido pendant groups. Bisnaphthalimide compounds have been shown to possess anticancer activity. The potential of this polymer to encapsulate, solubilize and enhance drug (5-fluorouricil and bis-(naphthalimidopropyl)-diaminooctane) cytotoxicity in BxPC-3 cells was evaluated. Our studies showed that the insoluble drugs could be formulated up to 4.3 mg mL^−1^ and 2.4 mg mL^−1^ inside the amphiphiles, respectively. Additionally, the novel poly(allylamine)-naphthalimide carrier resulted in an amplification of cytotoxic effect with drug treatment after 24 h, and was capable of reduction of 50% cell population at concentrations as low as 3 μg mL^−1^.

## 1. Introduction

Poor aqueous solubility or dissolution of active ingredients is one of the most significant problems hindering effective drug delivery. More than 40% of new drugs under investigation have poor water solubility and hence poor bioavailability [1,2]. A number of solubility-enhancing strategies including co-solvents, micronization and nanonisation, amorphous solid dispersions (ASDs) [3], co-crystal formation [4], surfactants [5], complexation utilizing cyclodextrins [6] and the use of polymers [7,8,9,10] have been utilized to address this problem.

Amphiphilic polymers have been developed as a first-rate alternative to low molecule weight surfactants for drug solubilisation. This is due to the lower excipient:drug ratios required for solubilisation and a higher degree of stability due to the decreased critical aggregation concentrations [11]. The most unique characteristic of amphiphilic polymers is their wide array of structures and architectures [11,12]. Amphiphilic polymers may exist as block copolymers [13], graft polymers [11], dendrimers [14] and star shaped polymers [15]. Each architecture has different physical properties, but all can be used to solubilise hydrophobic compounds.

Amphiphilic poly(allylamine) (PAA) derivatives have been explored for their potential in drug delivery [16,17,18,19]. Hoskins and colleagues assessed the in vitro and in vivo pancreatic anticancer action of a nano-sized novel poly(allylamine) derivative grafted with 5% cholesteryl pendant groups (CH_5_-PAA) for the formulation of a novel bisnaphthalimide compound [16]. Bisnaphthalimide based drugs act as DNA intercalators and have shown huge potential in pancreatic cancer therapy [16,20]. However, their activity is such that with increased drug potency, decreased drug solubility is observed. Hence careful formulation strategies are required in order to render these potent anticancer agents as clinically useable. Hoskins reported that after formulation into the CH_5_-PAA amphiphiles, bis(naphthalimidopropyl)diaminooctane (BNIPDaoct) resulted in solubility enhancement up to 0.3 mg mL^−1^ [16]. The CH_5_-PAA did not demonstrate any significant toxicity, yet the formulation demonstrated strong in vitro and in vivo anticancer action [16].

**Figure 1:**
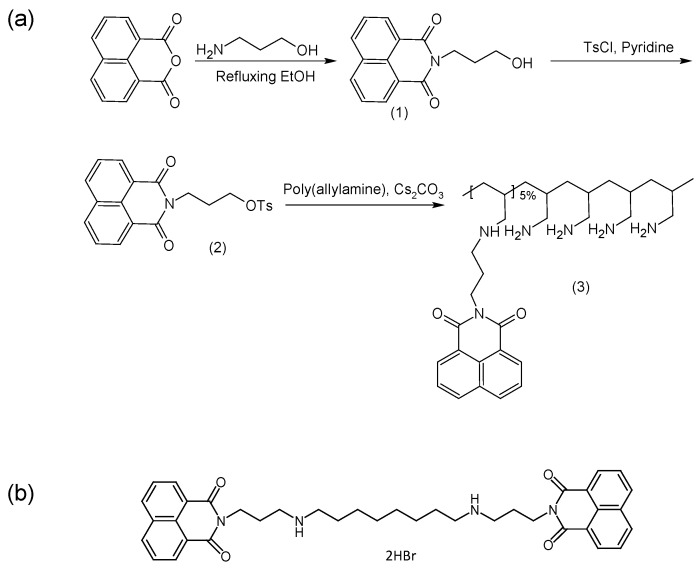
Chemical structures for (**a**) synthesis of poly(allylamine)-propylnaphthalimide (PAA-N) and (**b**) bis(naphthalimidopropyl)diaminooctane (BNIPDaoct).

Here we report the synthesis and evaluation of a novel poly(allylamine) (PAA) derivative which is grafted with 5% naphthalimide moieties (Figure 1a). The hydrophobic moieties used, are envisaged to allow for greater insertion and stability within the hydrophobic core through π-π interactions between the BNIPDaoct drug molecules (Figure 1b) and the planar naphthalimide pendant groups. Additionally, the use of the naphthalimide pendant groups will confer some inherent anti-cancer activity to the polymer itself. Ideally, for most drug delivery vehicles, biocompatibility is vital. However, in diseases such as pancreatic cancer, combined treatment and innovative therapies are required in order to overcome drug resistance and poor drug penetration. Nanotechnologies have shown to be useful for this. As such, we propose that a synergistic reduction in cell viability will occur after formulation in this system. It is proposed that these drug carriers if successful can be further functionalised with targeting moieties in order to bring the payload cargo to the exact site hence reducing likelihood of systemic toxicity.

The aggregation ability in aqueous environments and the ability to act as a drug solubilising agent was determined using novel BNIPDaoct and 5-fluorouricil (5-FU). 5-FU is an anti-cancer drug, which exerts its effects via inhibition of thymidylate synthase (TS) and the incorporation of its metabolites into RNA and DNA [21]. Numerous studies have demonstrated the potential use of 5-FU in pancreatic cancer as single therapy or in combination with the former gold standard treatment gemcitabine [22]. This study will evaluate whether the novel formulations formed, are capable of enhanced drug efficacy in vitro compared with the free drugs.

## 2. Results

### 2.1. Synthesis and Characterisation of Polymer

**Figure 2:**
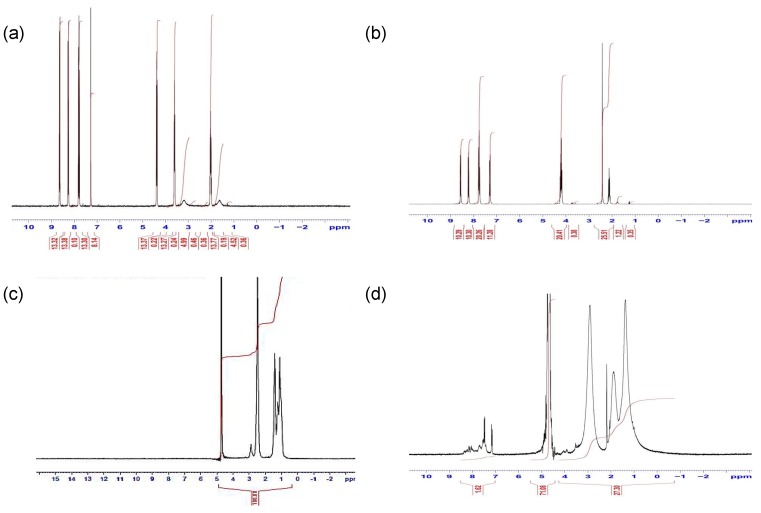
^1^H-NMR spectra of (**a**) compound 1 (Figure 1) in CDCl_3_ (**b**) compound 2 (Figure 1) in CDCl_3_, (**c**) PAA in D_2_O and (**d**) PAA-N in D_2_O carried out using 300 MHz NMR at 25 °C.

The structures of intermediates and products were analysed by ^1^H-NMR (Figure 2). Peak assignments for the ^1^H-NMR spectra of the naphthalimido alcohol product **1** were as follows: 2 ppm alkyl chain CH_2_, 3.6 ppm alkyl chain CH_2_ beside an O atom and O-H, 4.4 ppm alkyl chain CH_2_ beside a N atom and 7.8–8.6 ppm aromatic protons (Figure 2a). The O-tosyl spectrum (Figure 2b) showed assigned peaks at 2.2 ppm alkyl chain CH_2_, 2.5 ppm CH_3_ attached to an aromatic ring, 4.3 ppm alkyl chain CH_2_s beside O and N atoms and 7.3–8.5 ppm aromatic protons. The PAA showed characteristic peaks at 1.3 ppm CH_2_, 1.7 ppm CH, 2.2 ppm CH_2_ attached to -NH_2_ of PAA (Figure 2c). After modification with the naphthalimide group (PAA-propylnaphthalimide, Figure 2d) the spectrum showed additional aromatic proton peaks at 7.3–8.5 ppm, while the remaining propylnaphthalimide peaks were overlapped with the PAA peaks. The peak at 3.3 ppm was due to water.

The FTIR spectrum of compound **1**, (Figure S1a) displays a strong band at 3424 cm^−1^ that was assigned to the O-H stretch, in addition to a strong carbonyl group absorption at 1640 cm^−1^ which confirms the synthesis was successful. In the spectra for compound **2** the disappearance of the hydroxyl group band suggests the esterification between compound **1** and *para*-toluenesulfonyl chloride occurred (Figure S1b). The PAA spectra (Figure S1c) showed characteristic peaks at 3377 cm^−1^ and 2846 cm^−1^ due to the N-H and C-H in the polymer backbone. PAA-N (Figure S1d) possessed similar peaks to the PAA alone. However, the disappearance of strong peaks between 1410 cm^−1^ and 1177 cm^−1^, which related to the sulfonyl of the *para*-toluenesulfonyl group, confirms that the coupling had been successful.

### 2.2. Characterisation of Nano-Aggregates

**Figure 3:**
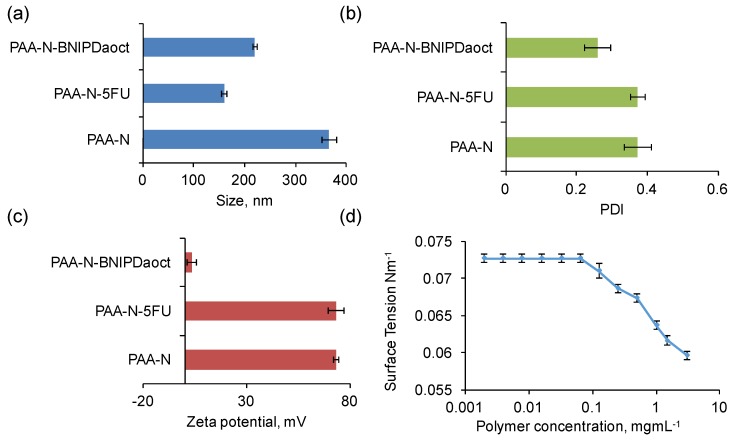
Characterisation of the nano-aggregates in aqueous media via (**a**) photon correlation spectroscopy and (**b**) polydispersity index, (**c**) zeta potential measurement (*n* = 3, ± SD) and (**d**) surface tension measurement.

Hydrodynamic diameter, polydispersity index (PDI) and zeta (ζ) potential were measured for the PAA-N aggregates in aqueous solution. The data showed that the self-assemblies formed possessed a hydrodynamic diameter of 366 ± 14 nm at 1 mg mL^−1^ (Figure 3a). The polydispersity of the aggregates formed was 0.37 (Figure 3b), this indicated that the aggregates formed were relatively mono-disperse in solution. The particle size (after storage in solution) was monitored over a 4-week period (Table S1), no significant change in size was determined rendering the particles stable over this timeframe. The amphiphiles possessed a positive surface charge in aqueous solution of +73 ± 1 mV (Figure 3c). This charge is attributed to the high primary amine content in the backbone of the PAA.

Surface tension measurement was used in order to determine the critical aggregation concentration (CAC) in aqueous media of the self-assemblies formed. The surface tension graph for PAA-N (Figure 3d) showed that the surface tension remains unchanged from that of water (0.00726 Nm^−1^) at very low concentrations (up to 0.065 mg mL^−1^). However, with increasing polymer concentration, surface tension decreased dramatically. The CAC value was determined from the surface tension graph to be 0.125 mg mL^−1^.

TEM imaging of the PAA-N (Figure S2a) showed nano-aggregates had been formed. The size of these aggregates was notably smaller compared to the photon correlation spectroscopy. This is due to the discrete differences in technique and measurement: photon correlation spectroscopy measures the hydrodynamic diameter in solution, which involves the interaction of the polymeric self-assemblies with the water molecules, whereas TEM allows for direct measurement of the aggregates in a solid state.

### 2.3. Drug Loading and Release

Drugs (5-FU and BNIPDaoct) were loaded into the core of the self-assemblies via probe sonication. The amount of drug encapsulated was quantified using UV-Vis spectroscopy and HPLC.

**Figure 4:**
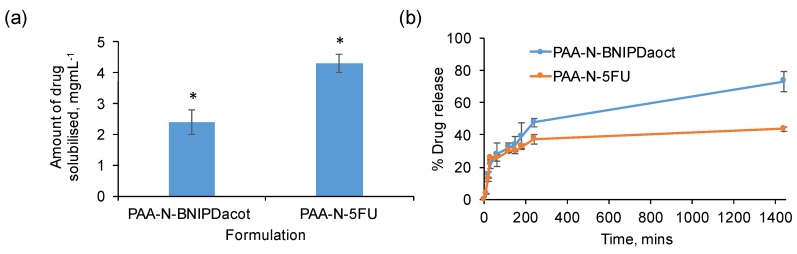
Drug loading (**a**) and release (**b**) studies of 5-FU and BNIPDaoct into and from PAA-N self-assemblies (*n* = 3, ± SD). * denotes a significant increase in aqueous solubility compared to the unformulated drug (*p* < 0.01). Studies were quantified using UV-Vis spectroscopy (5-FU) and HPLC (BNIPDoact).

Figure 4 shows that the PAA-N self-assemblies were capable of solubilizing 2.4 mg mL^−1^ and 4.3 mg mL^−1^ of BNIPDacot and 5-FU, respectively (Figure 4a). The difference in drug incorporation ability between the two drugs is possibly due to difference in molecular weight. The 5-FU exhibited an encapsulation efficiency of 17% whilst the BNIPDaoct was encapsulated at 10% into the PAA-N nano-aggregates. The 5-FU is a smaller molecule which may have resulted in greater quantities physically fitting within the core space of the nano-aggregates. The size of the nano-aggregates after drug loading was determined via photon correlation spectroscopy (Figure 3a) and TEM imaging (Figure S2a,b). Here the loaded particles appeared to undergo core compaction after drug loading with the hydrodynamic radius reducing from 366 nm of the PAA-N to 159 nm and 220 nm after drug incorporation (5-FU and BNIPDaoct respectively) (Figure 3a). This is likely due to the increase in hydrophobicity after drug incorporation pulling the pendant groups closer, resulting in a tightly packed compact aggregate. This trend was consistent with the smaller aggregates also being observed in the TEM micrographs (Figure S2b,c). Stability studies over a 4-week period indicated no change in particle size, as measured by photon correlation spectroscopy (Table S1).

It is not expected that drug loading into the core of polymeric self-assemblies should result in a change in zeta potential measurement. This is due to the fact that the surface charge of the aggregates should remain unchanged if a drug has been successfully incorporated into the core. As evident in Figure 3c, incorporation of 5-FU into the PAA-N did not result in any significant changes in surface charge. However, the data from the BNIPDaoct loaded PAA-N aggregates showed a dramatic reduction in surface charge from +73 mV to +3.6 mV. The reason for this is not known. The BNIPDaoct is a polyamine-based drug and hence when protonated should have an overall net positive charge and, therefore, should not form charge-charge complexes with the cationic PAA-N. However, the naphthalimide moieties at each end of the polyamine chain do carry residues with a high electron density. Therefore it may be possible that the drug molecules BNIPDaoct, have become anchored into the self-assemblies with one of the naphthalimido groups of the drug sitting exterior to the core. Further investigation would be needed to confirm this theory.

Drug release studies (Figure 4b) were carried out in water at room temperature over 24 h. The data showed that the BNIPDaoct loaded aggregates discharged their drug payload more rapidly than the 5-FU loaded aggregates. Both formulations experienced 25% drug release within the first hour. After 24 h the BNIPDaoct had released 73% of drug compared with only 43% of the 5-FU being released. This may have been due to the internalization mechanism of the drugs. Theoretically, the BNIPDaoct would have been expected to have been retained within the hydrophobic core of the self-assemblies for much longer than the 5-FU. This is due to the greater degree of hydrophobicity of the BNIPDaoct compared with the 5-FU as evidenced by their respective intrinsic solubilities. Additionally, it was postulated that upon inserting within the hydrophobic core, the BNIPDaoct would be able to stack in close proximity with little steric hindrance due to the planar nature of the molecule. However, given the large decrease in zeta potential observed (Figure 2a) after BNIPDaoct incorporation, this may not be the case, and hence, the more rapid drug release compared to the smaller 5-FU molecule. This finding does support the suggestion that polymer-drug complexation is occurring which is more rapidly broken down compared to the time taken for 5-FU to become un-encapsulated.

### 2.4. Cytotoxic Activity

**Figure 5:**
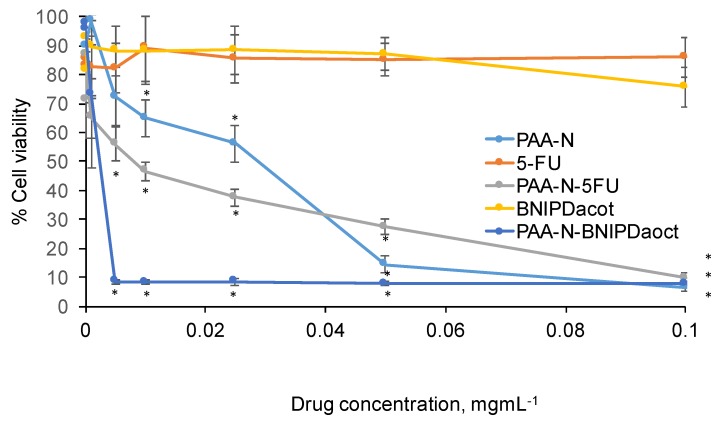
Cytotoxicity of 5-FU and BNIPDaoct loaded PAA-N in BxPC-3 cells after 24 h exposure (*n* = 3, ± SD). * denotes a significant reduction in viability compared with the control cells (*p* < 0.01).

The cytotoxic effect of PAA-N, BNIPDaoct, 5-fluorouracil, PAA-N-BNIPDaoct and PAA-N-5FU was determined by MTT assay in BxPC-3 cells after 24 h exposure (Figure 5). The data showed that both free drugs did not result in any significant decrease in cell viability over the concentrations tested.

The PAA-N carrier possessed an IC_50_ value of approximately 28 μg mL^−1^ which was reduced to 7.5 μg mL^−1^ after 5-FU incorporation. The BNIPDaoct formulation was more potent with IC_50_ observed at 3 μg mL^−1^ (10-fold). Interestingly, 0.05 mg mL^−1^ the PAA-N-5FU appeared less toxic than the empty carrier (PAA-N). However, upon further increase in concentration this appeared similar.

### 2.5. Cellular Uptake of Drug

**Figure 6:**
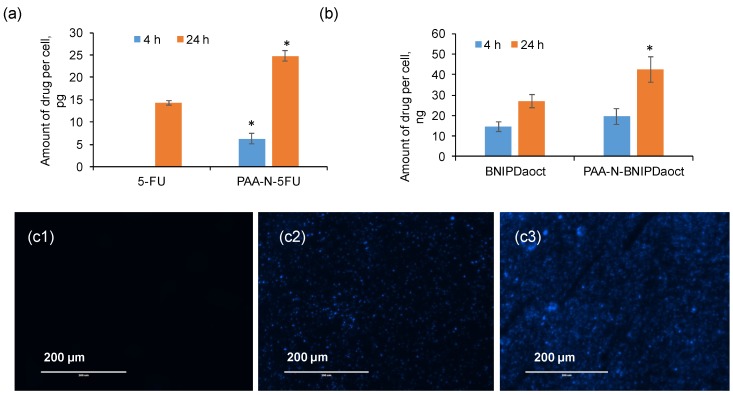
Intracellular drug uptake of PAA-N loaded aggregates with (**a**) 5-FU and (**b**) BNIPDaoct into BxPC-3 cells exposed to 50 μg mL^−1^ for 4 h and 24 h measured by UV-Vis spectroscopy and HPLC respectively (*n* = 3, ± SD). * denotes significant increase compared with the free drug (*p* < 0.01). (**c**) Fluorescence microscopy of PAA-N-BNIPDaoct internalised within BxPC-3 cells: 1: control, 2: 4 h incubation and 3: 24 h incubation.

Drug uptake in BxPC-3 cells was measured quantitatively using UV-Vis spectroscopy (5-FU) and HPLC (BNIPDoact) allowing for the amount of drug per cell to be estimated. Figure 6a shows the amount of 5-FU internalised into the cells, here it is evident that the 5-FU encapsulated within the PAA-N resulted in significantly greater (*p* < 0.001) uptake compared to the free drug. The BNIPDaoct resulted in greater cellular internalisation (Figure 6b) compared with the 5-FU. The enhancement of cellular internalisation due to the polymer encapsulation was not as pronounced in these studies. However, the intrinsic solubility of the free drug was so low that in order to administer to the cells, DMSO was required to solubilise the drug. This ability to become solubilised in organic solvents, means the BNIPDaoct is more likely to permeate well into the cells without the use of excipients—however, in order to be administered intravenously in patients, this highly insoluble drug does need to be solubilised initially.

Cellular uptake of the BNIPDaoct both free and in formulation can be observed in Figure 6c and Figure S3. Interestingly the polymer alone did not result in a fluorescent signal despite the presence of the naphthalimido moieties in its backbone (Figure S3(a1,a2)). This could be due to two reasons (i) The polymer concentration is so low (below the detection limit) and hence the relative quantity of the fluorophore is negligible, or (ii) the self-assembly structure of the hydrophobic naphthalimide pendant moiety into the core of the aggregate has shielded its fluorescence ability. This phenomenon was observed after both 4 h (Figure S3(a1)) and 24 h (Figure S3(a2)).

A further interesting phenomenon was observed which did not match with the HPLC data for cellular internalisation: After both 4 h and 24 h images of the drug alone (Figure S3(b1,b2)) showed minimal fluorescence compared with the polymer loaded samples (Figure 6(c2,c3)). The reason for this is not known. However, the general trend shows greater internalisation after incorporation into nanoparticles and is likely to be due to the difference in cellular internalisation mechanism whereby, consistently across a breadth of studies, intracellular drug concentrations are increased after incorporation into or onto nanoparticles [23,24,25,26]. This is reportedly due to the internalisation of nanoparticle vehicles via endocytosis, a phenomenon rarely reported for single drug molecules. Instead, the drug molecules are likely to enter the cells via the tight junction or other diffusion driven pathways with often take longer compared with endocytosis. However, the reason that this was not observed in the quantitative study is not known (and perhaps is explained by instrument sensitivity), although these findings do agree with the cytotoxicity data trend.

## 3. Discussion

This study highlights the potential of a naphthalimide grafted polyallylamine as a dual functioning platform for increased efficacy in pancreatic cancer cell lines. Here, we reported the successful synthesis and characterisation of the comb shaped polymer. We have demonstrated its ability to form nano-self assemblies capable of incorporation of hydrophobic drug entities into their lipophilic core. Poly(allyamine) (PAA)-based comb polymers have been reported previously to act as universal drug solubilising agents [27]. In particular to formulate novel bisnaphthalimide molecules. One previous study reported 0.3 mg mL^−1^ solubility of BNIPDaoct within PAA-Ch_5_ [16]. In another study the PAA was modified with a hydrophobic oxadiazole pendant group which resulted in 9.88 mg mL^−1^ BNIPDaoct solubilisation [18]. This highly potent drug has proven effective in vitro as a potential chemotherapy agent, however, clinical usage is severely hindered by its almost negligible solubility. Previous solubility studies showed some enhancement; however, the relatively low solubilisation potential results in higher excipient:drug ratios and, hence, more expensive therapies [16,18]. In this study we have shown that by substituting the bulky cholesteryl and oxadiazole moieties used previously with a planar naphthalimido moiety, greater quantity of drug compound can be introduced into the hydrophobic core in a ‘like dissolves like’ manner. Additionally, we believe that the planar nature of functionalities within the formulation may make the resultant nano-aggregates highly stable in solution as observed in our stability measurements. Interestingly, we observed that loading both anticancer drugs 5-FU and BNIPDaoct into the core resulted in particle compaction. This may be due to the planar nature of both the drug and formulation allowing for a less sterically hindered system, whereby drug molecules sit closer together forming a hydrophobic ‘strong-hold’. Such size phenomena has previously been reported elsewhere [28,29]: polymeric micelles experienced size reduction upon drug loading, which was explained by a lowering in aggregation number, erosion as well as hydrolysis [28,29]. Using our system, we were able to solubilise up to 2.4 mg mL^−1^ of BNIPDaoct which was an 8-fold improvement on previous findings.

Previous reports on the use of aromatic pendant groups attached to PAA had demonstrated a level of intramolecular and intermolecular aggregations [27]. In this study, we only observed one aggregation mechanism. This may be due to the smaller size of the naphthalimido moieties not harnessing enough hydrophobicity to initiate intramolecular aggregation so, therefore, relying solely on intermolecular aggregation where more than one polymer strand is required for nano self-assembly.

The zeta potential measurement after 5-FU loading was expectedly similar to the unloaded vehicle. This finding agrees with the principle that if the drug inserts fully into the hydrophobic core, it will become shielded from the exterior environment—which includes all of its charges and ability to interact. However, for BNIPDaoct the zeta potential measurement obtained after sonication was curious. Here, the data appeared to show a dramatic shift in zeta potential. Previously, PAA amphiphiles have been shown to be effective complexing agents for biological molecules such as insulin and salmon calcitonin [30,31,32]. Here, the charge-charge interactions initiate complex formation and hold the constituents tightly into the nanoparticles. It is possible that complex formation occurred between the BNIPDaoct, however, we do not think that is likely due to the polyamine chain resulting in a net positive charge on the molecule, a phenomena which we have previously been able to exploit for attachment onto negatively charged gold nanoparticle surfaces [20]. Hence, it may be the case that the linear drug molecules are either experiencing one of the naphthalimido moieties in their structure anchoring into the lipophilic self-assembly core with the other directed towards the surface. Alternatively, the naphthalimido moieties are anchored into different cores of two self-assemblies hence stitching them together—therefore the drug molecules are sitting more towards the surface of the macromolecules. Looking at the irregular morphology of these aggregates in the TEM (Figure S2c) compared with those loaded with 5-FU (Figure S2b) this may be likely. However, further investigation is required to confirm such a hypothesis.

This polymer was intentionally designed not only to allow greater insertion of drug molecules into its core compared with our previous system [16], but also to possess its own inherent cytotoxic nature. This may seem counterintuitive for a drug delivery vehicle. However, in diseases such as pancreatic cancer, extremely harsh therapies are required in order to eradicate the rapidly proliferating cells. We believe that the more stable particulates formed will result in less breakdown, premature release of drug and also exposure of hydrophobic pendant group to the external environment. This study serves as a proof of concept to elucidate whether the naphthalimide moieties could result in an increase in therapeutic effect in combination with the bisnaphthalimide drugs. This study resulted in a drug formulation exhibiting an IC_50_ of 3 µg mL^−1^ against pancreatic cancer cells. In previous studies [16] with the same drug we had achieved slightly lower IC_50_ values of 0.7 µg mL^−1^, however, this was after 48 h drug exposure and not 24 h as in this work. Additionally, in previous reports we have demonstrated no observable IC_50_ for the previous gold standard treatment gemcitabine in BxPC-3 cells after 24 h [20], showing that this formulation is notably more rapid in its potency. Therefore, we believe there is some potential in our findings and currently further investigations are underway in order to determine whether this formulation may be modified in a manner which will allow for safe nanoparticle travel in the bloodstream before actively seeking out the pancreatic tumours. We must first address the timeliness of drug release in the context of application and time for intracellular tracking. We will do this by exploiting active targeting mechanisms alongside exploitation of the highly specific flora within the tumour microenvironment in order to render a stimuli responsive system. In addition, further in vitro evaluation is required in non-cancerous cell lines in order to estimate the extent of toxicity to health tissue which will help inform the surface engineering of non-toxic polymers anchored onto the nano-aggregate surface to render more effective biocompatibility.

## 4. Materials and Methods

### 4.1. General Information

Poly(allylamine) (PAA) (MW:17,500), 1,8-naphthalic anhydride, 3-aminopropan-1-ol, *para*-toluenesulfonyl chloride and caesium carbonate were purchased from Alfa Aesar (Heysham, UK). Chloroform, diethyl ether, ethanol and anhydrous pyridine were purchased from Fisher Scientific (Loughborough, UK). Deuterated chloroform, deuterated dimethyl sulfoxide and deuterium oxide were purchased from Apollo Scientific Limited (Stockport, UK). Human pancreatic adenocarcinoma (BxPC-3) cells were obtained from ATCC (Manassas, VA, USA). Foetal bovine serum, Roswell Park Memorial Institute Medium (RPMI) 1640, trypsin and penicillin streptomycin were purchased from Life Technologies (Runcorn, UK).

### 4.2. Synthesis and Characterisation of Polymer

#### 4.2.1. Reaction of 1,8-Naphthalic Anhydride with 3-Aminopropan-1-ol: Synthesis of *N*-(3-Hydroxy-propyl)Naphthalimide (**1**)

1,8-Naphthalic anhydride (3.96 g, 0.02 mmol) was suspended in ethanol (100 mL) in a 250 cm^3^ round bottom flask. The suspension was stirred using a magnetic stirrer and bar. 3-Aminopropan-1-ol (1.5 g, 0.02 mmol) was added to the mixture dropwise. The mixture was heated to reflux for 15 h and was then allowed to cool. A small amount of ethanol was removed using a rotary evaporator until a solid precipitate began to form. The mixture was then cooled in the refrigerator (3–5 °C) until no more precipitate formed. The mixture was filtered under vacuum (Buchner funnel and flask) and the pale cream crystalline precipitate was dried on the filter. The dried precipitate was crystallised from ethanol, filtered and the crystalline product dried in vacuum at 60 °C to give pale cream-coloured needles.

#### 4.2.2. Tosylation of *N*-(3-Hydroxypropyl)Naphthalimide (**1**): Synthesis of 3-tosyloxypropyl) naphthalimide (**2**)

*N*-(3-Hydroxypropyl)naphthalimide (**1**, 2.55 g, 10 mmol) was dissolved in anhydrous pyridine (dried over solid KOH) (80 mL) in a dry 250 cm^3^ round bottom flask. The reaction solution was left to stir at 0 °C for 10 min. After this time *para*-toluenesulfonyl chloride (2.86 g, 15 mmol) was added dropwise over 30 min. The mixture was then stored at 4 °C in a refrigerator overnight. The refrigerated mixture was poured slowly onto 200 mL icy water in a 500 mL beaker, followed by vigorous stirring to deliver a viscous liquid that solidified quickly on cooling. The solid was filtered through a Buchner funnel and washed with water several times. The dried precipitate was crystallised from ethanol, filtered and the crystalline product dried in vacuum at 60 °C to give a white powdery precipitate.

#### 4.2.3. Liberation of PAA Free Base

PAA-HCl salt (10 g) was dissolved in deionised water. Sodium hydroxide (8 g) was added into the solution until pH 13 was achieved. The mixture was subsequently stirred for 1 h. After this time the PAA solution was exhaustively dialysed against water using 7000 Dalton Visking membrane for 24 h. The resultant solution was freeze-dried to obtain a white solid.

#### 4.2.4. Synthesis of PAA-Propylnaphthalimide (**3**)

PAA (1 g) was dissolved in dissolved in 1:1 (*v*/*v*) methanol: chloroform (30 mL) with stirring. *N*-(3-tosyloxypropyl)naphthalimide (0.605 g) was added to solution followed immediately by caesium carbonate (1.2 g) and the mixture was stirred at room temperature for 24 h. The solvent was removed using a rotary evaporator and the polymer residue was washed three times with diethyl ether and subsequently dried. The product was purified by dissolving in water and exhaustive dialysis (Visking membrane, molecular weight cut-off = 12–14 kDa) against deionized water (3 L) with six changes over 24 h. The resultant solution was freeze-dried.

### 4.3. Characterisation of Self-Assemblies

Self-assemblies were formed by dissolving solid polymer in water. The solutions were probe sonicated for 5 min before filtration through a 0.45 μm syringe filter.

#### 4.3.1. Characterisation Using Photon Correlation Spectroscopy and Zeta Potential Measurement

Nano-aggregate solutions/formulations were formed in water and analysed for their size and surface charge. The size was monitored over a 4-week period. Hydrodynamic diameter, polydispersity index (PDI) and zeta (ζ) potential measurements were performed using PCS on a Zetasizer Nano-ZS (Malvern Instruments, Malvern, UK). The data presented are the averaged values of three successive measurements.

#### 4.3.2. Transmission Electronic Microscopy (TEM) Imaging

Transmission electron microscopy (TEM) was used to visualise the polymer formulas. Imaging was performed and processed using a JEOL 1200 EX-FDL5000 microscope (Jeol, Tokyo, Japan) transmission electron microscope. TEM samples were prepared by placing one drop of the formulations prepared as described above, onto formvar/carbon-coated 200 mesh nickel grids and dried under a heat lamp for 3 h.

#### 4.3.3. Surface Tension Measurement

The surface tension of polymer formulation was investigated by a torsion balance (Torsion Balance Supplies, Weston-super-Mare, UK). In brief, various concentrations (0.00195–3 mg mL^−1^) of naphthalimide-PAA were prepared in deionised water. Samples were sonicated for 5 min using a probe sonicator the samples were cooled to room temperature, after which the surface tension was measured. All experiments were run in triplicate. Critical aggregation concentration was indicated by dramatic reduction in surface tension, as observed by inflection in the graph.

### 4.4. Drug Loading

Polymer was dissolved in deionised water (1 mg mL^−1^) and probe sonicated for 10 min using a Soniprep 150 (Wolflabs, Pocklington, UK). The hydrophobic drug (5 mg mL^−1^) of was added individually at 5:1 initial drug:polymer weight ratio and the drug-polymer solutions were probe sonicated for a further 10 min. The polymer solutions were filtered using a 0.45 µm syringe filter. The drug content was quantified: 5-FU by UV-visible spectroscopy at 256 nm (in DMSO) and BNIPDaoct using the reverse phase high performance liquid chromatography (HPLC) coupled to a fluorescent detector using a RP Zorbax ODS 250 mm × 46 mm × 5 µm HPLC column (Hichrom, Lutterworth, UK). The mobile phase was 55:45 (*v*/*v*) buffer:acetonitrile and the flow rate was 1 mL min^−1^. The buffer for the mobile phase was made up of 0.432 g octane sulfonic acid and 1.64 g anhydrous sodium acetate made up to 200 mL with deionised water, which the solution was subsequently pH adjusted to pH 4.5. The flow rate was set at 1 mL min^−1^, 20 µL injection volume and excitation and emission wavelengths set to 234 nm and 294 nm respectively. Control samples of polymer alone were run and subtracted from the data in order to eliminate any fluorescence due to the naphthalimide residues within the intrinsic polymer structure (for the BNIPDaoct formulation samples). The solutions were compared to calibrations of their respective drugs (R^2^ = 0.999 for 5-FU and R^2^ = 0.998 for BNIPDaoct), all experiments were run in triplicate. Encapsulation efficiencies (EE) were calculated to be: % EE = (drug concentration determined by HPLC/original drug concentration) × 100%.

### 4.5. Drug Release

Formulations (2 mL) were pipetted into Visking membrane of 12–14 KDaltons and dialysed against deionised water for 24 h. At set time intervals (e.g., 1 min, 5 min, 10 min etc.), 1 mL of water was removed and drug content analysed as previously described. The cumulative drug release profile was calculated in respect to drug loading concentration. All experiments were run in triplicate.

### 4.6. Cytotoxic Activity

For evaluation of the cytotoxic potential of PAA-N: drug aggregates, an MTT assay was performed in comparison with PAA-N alone and free drugs, in equivalent weight as in PAA-N: drug aggregates) at 24 h incubation time. In brief, human pancreatic adenocarcinoma (BxPC-3) cells were cultured in RPMI medium containing 10% foetal bovine serum (FBS) and 1% penicillin streptomycin (P/S). Cells were sub-cultured into a 96-well plate at, and then the plates were incubated in the incubator at 37 °C in humidified 5% CO_2_ atmosphere. Cells were then treated with PAA-N alone, PAA-N-BNIDaoct, PAA-N-5FU, and free drugs BNIPDaoct and 5-fluorouracil (dissolved in DMSO—Stock solutions of 20 mg mL^−1^ were prepared and diluted with cell culture media). Once 70% cell confluence was achieved the media was removed and replaced with the drugs or formulations (0.00001–0.1 mg mL^−1^). Following this, the media was removed and replaced with a 100 μL solution of 10% MTT in media and incubated for 4 h. After which, the absorbance of the formazan solution is read spectrophotometrically at 570 nm and cell viability was calculated with respect to the controls. PBS and Triton X (80 µL each) were used as the negative and positive controls respectively. All experiments were run independently in triplicate.

### 4.7. Cellular Uptake of Drug

#### 4.7.1. Intracellular Drug Concentration

BxPC-3 cells were seeded at a density of (50,000 cells/well) into 6-well plates and incubated for 24 h. The media was then replaced with 50 µg mL^−1^ solution of formulation, or free drug as well as the polymer alone (at equivalent concentration) diluted in media. The plates were incubated for 4 h and 24 h at 37 °C. Following incubation, the media was removed and the cells were washed three times with PBS and trypsinised. The cell suspension was counted for viable cells and 100,000 cells transferred into an Eppendorf tube. Deionised water (1 mL) was added to lyse the cells and the tube was centrifuged at 500 rpm for 5 min in a Z-323 centrifuge (Hermule, Wehingen, Germany) to remove the cell debris. The supernatant was removed and diluted in DMSO for 5-FU and 55:45 (*v*/*v*) buffer: acetonitrile (previously described) for BNIPDaoct. Control samples of polymer alone were run and subtracted from the data in order to eliminate any fluorescence due to the naphthalimide residues within the intrinsic polymer structure (for the BNIPDaoct formulation samples). Drug content was measured as previously described and calculated per cell. All experiments were carried out in triplicate.

#### 4.7.2. Fluorescence Microscopy

Cell lines were seeded onto glass cover slip in 6-well plates (50,000 cells/well) and treated as described above. After washing, the cells were fixed with 2% paraformaldehyde for 10 min and further washed three times with PBS. The glass slips were fixed on the glass slides and visualised by fluorescence microscopy (Invitrogen, EVOS^TM^, Runcorn, UK).

## 5. Conclusions

This study highlights the potential of PAA-N as a drug solubilising agent in order to amplify the therapeutic effect in pancreatic cancer cell lines, particularly in combination with bisnaphthalimide-based anti-tumour drug molecules. These exciting findings will inform the second generation of such delivery systems whereby complete physiological protection of the drug molecule and polymer will be conferred until site specific delivery of drug is achieved. This will be realised through surface decoration with targeting moieties and additional stimuli responsive residues in order to ensure safety before reaching the target site.

## Supplementary Materials

The following are available online at http://www.mdpi.com/1424-8247/11/4/91/s1, Figure S1. FTIR spectra of (a) Compound (1), (b) Compound (2), (c) PAA and (d) PAA-N. Spectra an average of 64 scans. Figure S2. Transmission electron micrographs of (a) PAA-N, (b) PAA-N-5FU and (c) PAA-N-BNIPDaoct. Figure S3. Fluorescence microscopy of BNIPDaoct loaded PAA-N internalised within BxPC-3 cells after (a) 4 h and (b) 24 h with 1: PAA-N and 2: BNIPDaoct. Table S1. Size of nano-aggregates over 4-week period as measured by photon correlation spectroscopy.

## References

[1] Hoskins C., Papachristou A., Ho T.M.H., Hine J., Curtis A.D.M. (2016). Investigation into Drug Solubilisation Potential of Sulfonated Calix[4]Resorcinarenes. J. Nanomed. Nanotechnol..

[2] Zahari V., Petra D., Slavka T. (2018). Effect of Surfactant Molecular Structure on Progesterone Solubilisation. J. Drug Deliv. Sci. Technol..

[3] Teja S.B., Patil S.P., Shete G., Patel S., Bansal A.K. (2013). Drug-Excipient Behaviour in Polymeric Amorphous Solid Dispersions. J. Excipients Food Chem..

[4] Panzade P., Shendarkar G., Shaikh S., Rathi P.B. (2017). Pharmaceutical Cocrystal of Piroxicam: Design, Formulation and Evaluation. Adv. Pharm. Bull..

[5] Rangel-Yagul C.O., Pessoa Jnr A., Tavares L.C. (2005). Micellar Solubilization of Drugs. J. Pharm. Pharmaceut. Sci..

[6] Loftsson T., Brewster M.E. (1996). Pharmaceutical Applications of Cyclodextrins. 1. Drug Solubilization and Stabilization. J. Pharm. Sci..

[7] Lu Y., Park K. (2013). Polymeric Micelles and Alternative Nanonized Drug Delivery Vehicles for Poorly Soluble Drugs. Int. J. Pharm..

[8] Yu B.G., Okano T., Kataoka K., Kwon G. (1998). Polymeric Micelles for Drug Delivery: Solubilization and Haemolytic Activity of Amphotericin B. J. Control. Release.

[9] Kulthe S.S., Choudhari Y.M., Inamdar N.N., Mourya V. (2012). Polymeric Micelle: Authorative Aspects for Drug Delivery. Des. Monomers Polym..

[10] Xu W., Ling P., Zhang T. (2013). Polymeric Micelles, a Promising Drug Delivery System to Enhance Bioavailability of Poorly Water-Soluble Drugs. J. Drug Deliv..

[11] Hoskins C., Kong Thoo-Lin P., Cheng W.P. (2012). A Review on Comb-Shaped Amphiphilic Polymers for Hydrophobic Drug Solubilisation. Therapeut. Deliv..

[12] Rinkenauer A.C., Schubert S., Traeger A., Schubert U.S. (2015). The Influence of Polymer Architecture on In Vitro pDNA Transfection. J. Mater. Chem. B.

[13] Förster A., Markus A. (1998). Amphiphilic Block Copolymers in Structure Controlled. Adv. Mater..

[14] Choudhary S., Gupta L., Rani S., Dave K., Gupta U. (2017). Impact of Dendrimers on Solubility of Hydrophobic Drug Molecules. Front. Pharmacol..

[15] Ooya T., Lee J., Park K. (2003). Effects of Ethylene Glycol-Based Graft, Star-Shaped, and Dendritic Polymers on Solubilisation and Controlled Release of Paclitaxel. J. Control. Release.

[16] Hoskins C., Ouaissi M., Lima S.C., Cheng W.P., Loureirio I., Mas E., Lombardo D., Cordeiro-da-Silva A., Ouaissi A., Kong Thoo Lin P. (2010). In Vitro and In Vivo Anticancer Activity of a Novel Nano-Sized Formulation Based on Self-Assembling Polymers Against Pancreatic Cancer. Pharm. Res..

[17] Al-Shakarchi W., Alsuraifi A., Curtis A., Hoskins C. (2018). Dual Acting Polymeric Nano-Aggregates for Liver Cancer Therapy. Pharmaceutics.

[18] Barnett C.M., Martin L.R., Curtis A., Kong Thoo Lin P., Cheng W.P., Hoskins C. (2013). Poly(Allylamine) Magnetomicelles for Image Guided Drug Delivery. Pharmaceut. Nanotechnol..

[19] Zafar A., Pilkington L.I., Haverkate N.A., van Rensburg M., Leung E., Kumara S., Denny W.A., Barker D., Alsuraifi A., Hoskins C. (2018). Investigation into Improving the Aqueous Solubility of the Thieno[2,3-*b*]Pyridine Anti-Proliferative Agents. Molecules.

[20] Malekigorji M., Alfahad M., Kong Thoo Lin P., Jones S., Curtis A., Hoskins C. (2017). Thermally Triggered Theranostics for Pancreatic Cancer Therapy. Nanoscale.

[21] Harrap K.R., Jackman A.L., Newell D.R., Taylor G.A., Hughes L.R., Calvert A.H. (1989). Thymidylate Synthase: A Target for Anticancer Drug Design. Adv. Enzyme Reg..

[22] Wang W.B., Yang Y., Zhao Y.P., Zhang T.P., Liao Q., Shu H. (2014). Recent Studies of 5-Fluorouricil Resistance in Pancreatic Cancer. World J. Gastrenterol..

[23] Kou L., Sun J., Zhai Y., He Z. (2013). The Endocytosis and Intracellular Fate of Nanomedicines: Implication for Rational Design. Asian J. Pharm. Sci..

[24] Akinc A., Battaglia G. (2013). Exploiting Endocytosis for Nanomedicines. Cold Spring Harb. Perspect. Biol..

[25] Sahay G., Alakhova D.Y., Kabanov A.V. (2010). Endocytosis of Nanomedicines. J. Control. Release.

[26] Salatin S., Khosroushahi A.Y. (2017). Overviews on the Cellular Uptake Mechanism of Polysaccharide Colloidal Nanoparticles. J. Cell. Mol. Med..

[27] Hoskins C., Kong Thoo Lin P., Tetley L., Cheng W.P. (2012). Novel Fluorescent Amphiphilic Poly(Allylamine) and Their Supramolecular Self-Assemblies in Aqueous Media. Polym. Adv. Technol..

[28] Sharma P.K., Bhatia S.R. (2004). Effect of Anti-Inflammatories on Pluronic F127: Micellar Assembly, Gelation and Partitioning. Int. J. Pharm..

[29] Basak R., Bandyopadhyay R. (2013). Encapsulation of Hydrophobic Drugs in Pluronic F127 Micelles: Effects of Drug Hydrophobicity, Solution Temperature, and pH. Langmuir.

[30] Thompson C.J., Cheng W.P., Gadad P., Skene K., Smith M., Smith G., McKinnon A., Knott R. (2001). Uptake and Transport of Novel Amphiphilic Polyelectrolyte-Insulin Nanocomplexes by Caco-2 Cells-Towards Oral Insulin. Pharm. Res..

[31] Cheng W.P., Thompson C., Ryan S.M., Aguirre T., Tetley L., Brayden D.J. (2010). In Vitro and in vivo Characterisation of a Novel Peptide Delivery System: Amphiphilic Polyelectrolyte-Salmon Calcitonin Nanocomplexes. J. Control. Release..

[32] Thompson C.J., Uchegbu I.F., Tetley L., Cheng W.P. (2005). The Use of Novel Combed Shaped Amphiphilic Polymers for Oral Delivery of Insulin. Int. J. Pharm..

