# Supplementary material for: A Novel PAA Derivative with Enhanced Drug Efficacy in Pancreatic Cancer Cell Lines
Source: Pharmaceuticals (Basel). 2018 Sep 22;11(4):91. doi: 10.3390/ph11040091 (PMC6315666; doi:10.3390/ph11040091)
Supplement: Supplementary file 1 [file pharmaceuticals-11-00091-s001.pdf]

Supplementary Information

A Novel PAA Derivative with Enhanced Drug Efficacy in Pancreatic Cancer Cell Lines

Ali Alsuraifi [1,2], Paul Kong Thoo Lin [3], Anthony Curtis [1], Dimitrios A. Lamprou [4,*] and Clare Hoskins [1,*]

1 Institute of Science and Technology in Medicine, Keele University, Keele ST5 5BG, UK; a.t.y.alsuraifi@keele.ac.uk (A.A.); a.d.m.curtis@keele.ac.uk (A.C.)
2 College of Dentistry, University of Basrah, Basrah 61004, Iraq
3 School of Pharmacy and Life Sciences, Robert Gordon University, Aberdeen AB10 7GJ, UK; p.v.s.kong-thoo-lin@rgu.ac.uk
4 School of Pharmacy, Queen’s University Belfast, Belfast BT9 7BL, UK
* Correspondence: d.lamprou@qub.ac.uk (D.A.L.); c.hoskins@keele.ac.uk (C.H.); Tel.: +44-28-9097-2617 (D.A.L.); +44-17-8273-4799 (C.H.)

(a)

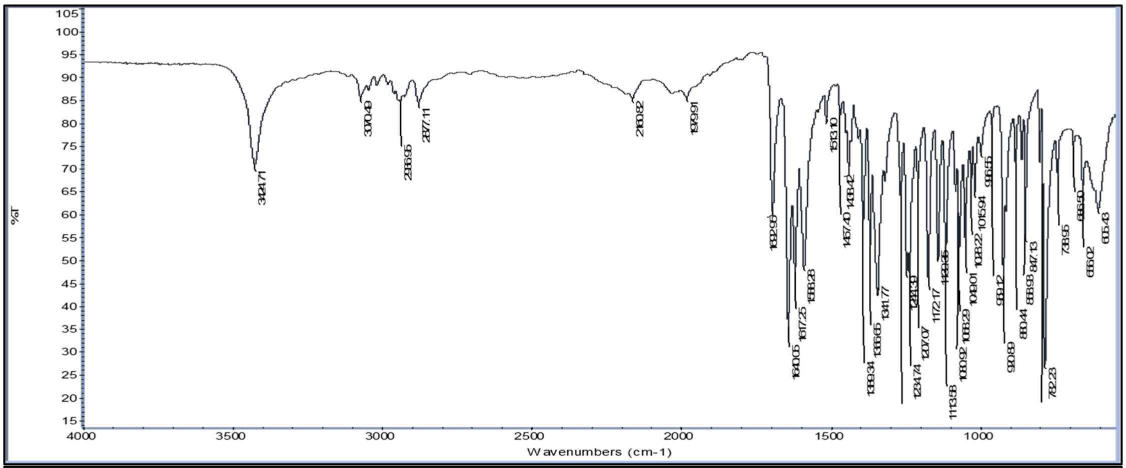

(b)

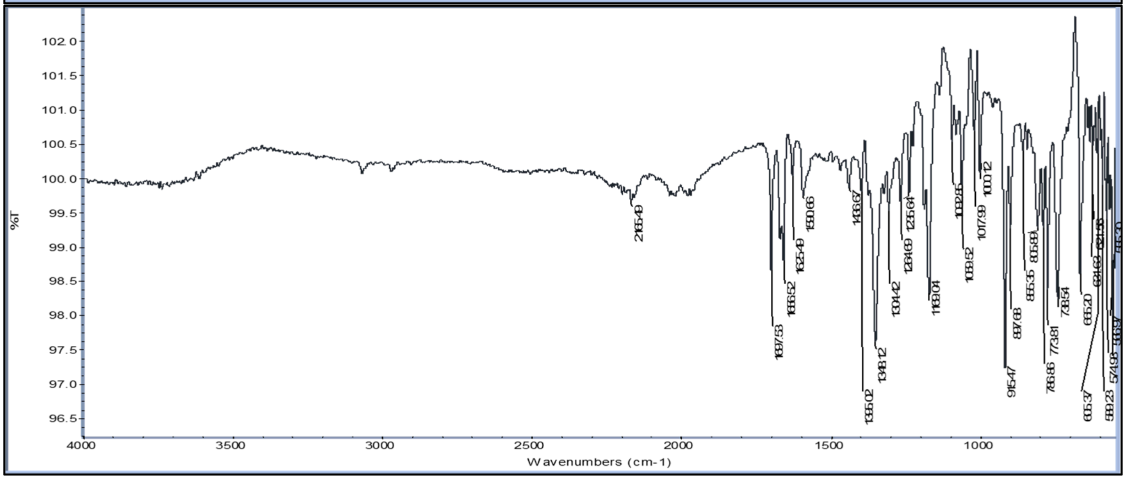

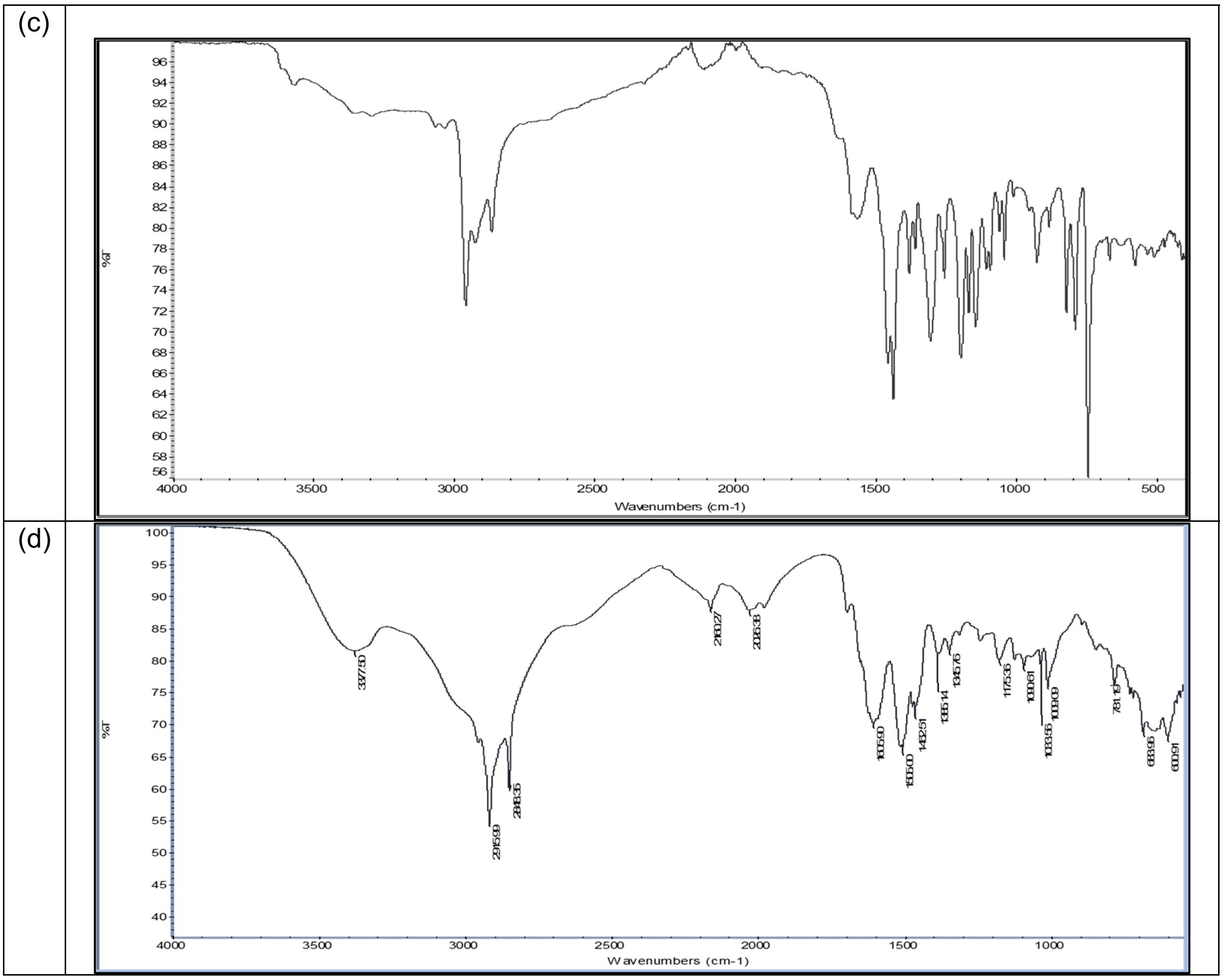

Figure SI-1. FTIR spectra of (a) Compound **1,** (b) Compound **2**, (c) PAA and (d) PAA-N. Spectra are averages of 64 scans.

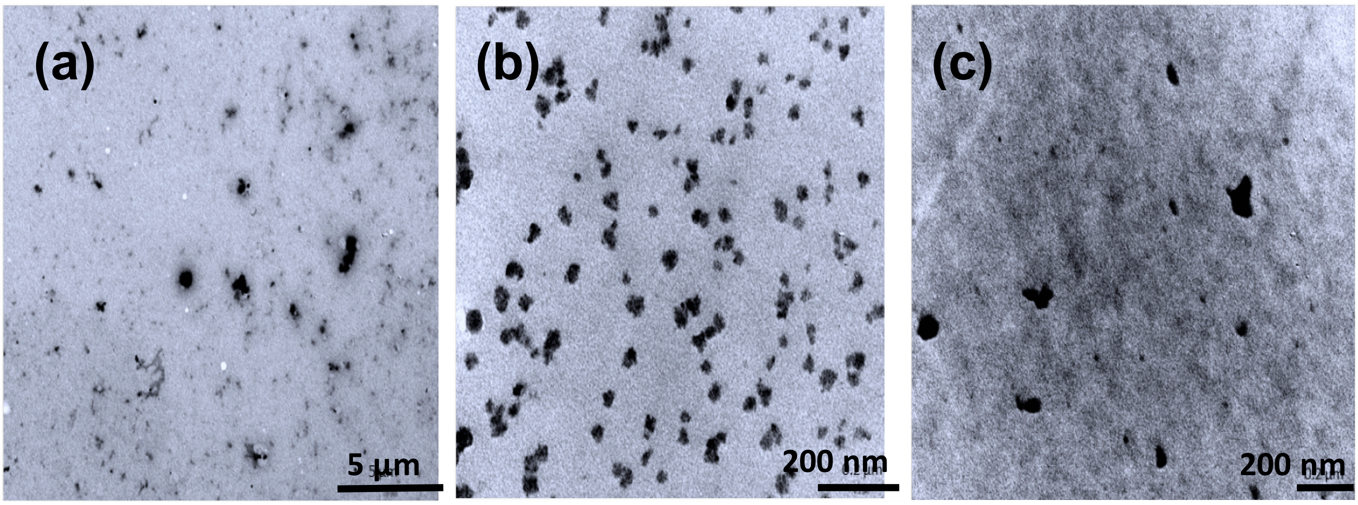

Figure SI-2. Transmission electron micrographs of (a) PAA-N, (b) PAA-N-5FU and (c) PAA-N-BNIPDaoct.

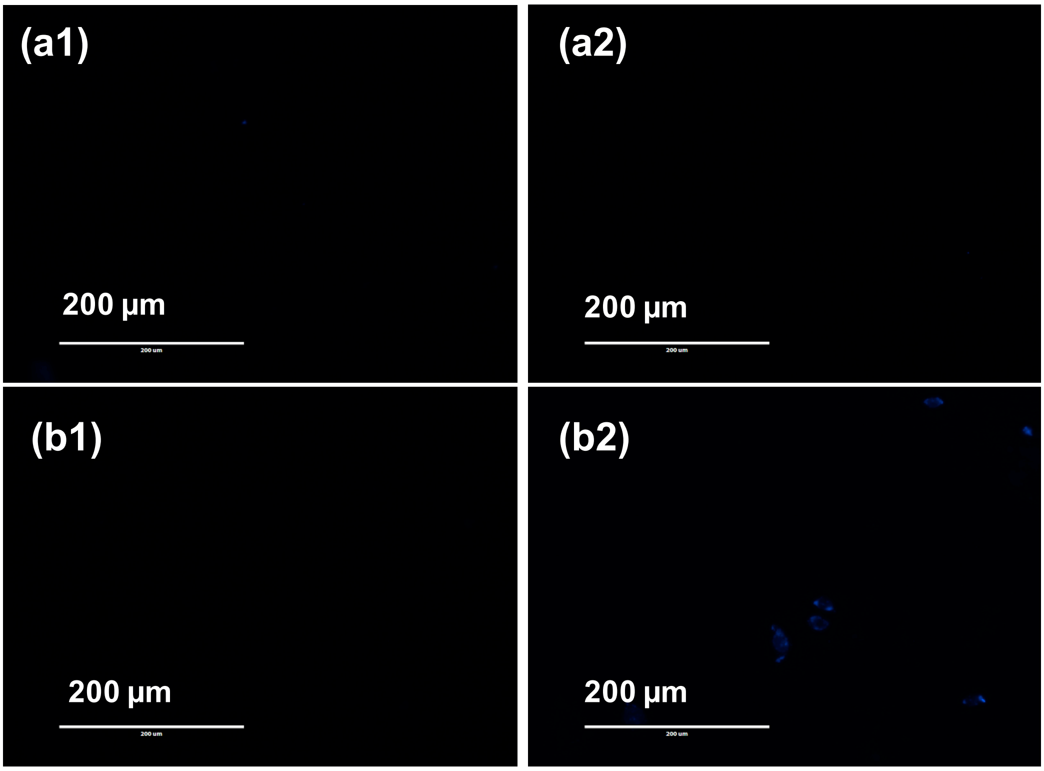

Figure SI-3. Fluorescence microscopy of BNIPDaoct loaded PAA-N internalised within BxPC-3 cells after (a) 4 h and (b) 24 h with 1: PAA-N and 2: BNIPDaoct.

Table SI-1. Size of nano-aggregates over 4-week period as measured by photon correlation spectroscopy.

	Time, weeks				
	t=0	t= 1	t=2	t=3	t=4
	Size, nm (SD)	Size, nm (SD)	Size, nm (SD)	Size, nm (SD)	Size, nm (SD)
PAA-N	367 (14)	374 (12)	365 (12)	366 (25)	368 (2)
PAA-N-5FU	159 (2)	152 (8)	157 (8)	156 (4)	158 (10)
PAA-N-BNIPDaoct	220 (3)	220 (10)	227 (4)	220 (7)	220 (6)
